# Person‐Centred Nursing in Allogeneic Stem Cell Transplantation Using a Conversation Tool: A Qualitative Study

**DOI:** 10.1111/scs.70153

**Published:** 2025-11-07

**Authors:** Cecilia Engberg de Carvalho, Anna O'Sullivan, Karin Bergkvist, Carina Lundh Hagelin, Jeanette Winterling, Annika Malmborg Kisch

**Affiliations:** ^1^ Department of Health Sciences Lund University Lund Sweden; ^2^ Department of Haematology, Oncology and Radiation Physics Skåne University Hospital Lund Sweden; ^3^ Department of Health Care Sciences Marie Cederschiöld University Stockholm Sweden; ^4^ Department of Nursing Sciences Sophiahemmet University Stockholm Sweden; ^5^ Department of Public Health and Caring Sciences Uppsala Sweden; ^6^ Department of Neurobiology, Care Sciences and Society, Division of Nursing Karolinska Institutet Stockholm Sweden; ^7^ Karolinska Comprehensive Cancer Centre, Medical Unit HHLH Karolinska University Hospital Stockholm Sweden

**Keywords:** allogeneic haematopoietic stem cell transplantation, cancer rehabilitation, needs assessment, nurse–patient communication, person‐centred care, person‐centred nursing, qualitative research

## Abstract

**Background:**

Patients undergoing allogeneic haematopoietic stem cell transplantation (allo‐HCT) often face complex and evolving needs throughout recovery. Person‐centred nursing (PCN) is essential in this context yet remains underexplored in specialised care settings. In Sweden, the Assessment of Rehabilitation Needs Checklist (ARNC) is commonly used in cancer care, but its role in supporting person‐centred conversations has not been investigated.

**Aim:**

The aim of this study was to investigate how the use of the ARNC as a conversation tool promotes PCN within the allo‐HCT context.

**Methods:**

This qualitative study was conducted at two major allo‐HCT centers in Sweden. Data were collected through semi‐structured interviews with patients (*n* = 16), focus group discussions with registered nurses (RNs, *n* = 16), and from 30 memos written by RNs. Reflexive thematic analysis was used.

**Results:**

Three overarching themes were developed: (1) *Letting the Story Emerge*, (2) *Unmet Needs* and (3) *Structural Gaps in Practice*. The ARNC facilitated individualised conversations and helped identify unmet needs, including sensitive or previously unvoiced concerns. However, the lack of follow‐up and organisational constraints, such as time pressure and fragmented care settings, limited its capacity to support shared care planning and sustained engagement.

**Conclusion:**

When used in dialogue, the ARNC has the potential to support person‐centred nursing in allo‐HCT by enabling narrative‐based, needs‐driven conversations. However, its effectiveness depends on structured follow‐up and organisational conditions that promote relational care.

## 1 Introduction

Patients undergoing allogeneic haematopoietic stem cell transplantation (allo‐HCT) face a demanding and often unpredictable care trajectory, characterised by physical symptoms, emotional strain and psychosocial or existential concerns [1, 2]. Chronic graft‐versus‐host disease (GvHD), persistent fatigue, or fear of relapse often persist long after the acute treatment phase [3, 4, 5]. These challenges place high demands on both patients and family caregivers, calling for approaches that are flexible, responsive and attentive to individual experiences [6, 7]. Encompassing both physical and psychosocial support, person‐centred rehabilitation has been shown to alleviate symptoms and promote adjustment [8]. Recovery after allo‐HCT rarely follows a linear path but unfolds in distinct phases, with shifting patient needs [9]. Therefore, structured assessments are vital to guide care and support conversations about what matters most [10, 11].

Person‐centeredness is widely promoted in healthcare, supported by international guidelines and growing evidence [12, 13]. Despite broad endorsement, person‐centeredness is variably defined and implemented, particularly in complex or high‐intensity settings [14]. Person‐centeredness emphasises a holistic approach that acknowledges the person behind the patient. It involves recognising individual values, supporting autonomy and promoting collaborative relationships between patients and professionals [15, 16]. Within nursing, these values have been further developed into a relational and reflective approach to practice. Person‐centred nursing (PCN) integrates these principles into daily care interactions between patients and registered nurses (RNs), drawing on clinical knowledge, ethical awareness and sensitivity to what matters to each individual [17, 18].

The PCN framework by McCormack and McCance [19] outlines how person‐centred values can be embedded in practice across four interdependent domains: (1) RNs' personal and professional attributes (prerequisites), (2) the organisational and relational conditions that shape care delivery (care environment), (3) the interactions through which person‐centeredness is enacted (person‐centred processes), and (4) the resulting outcomes in terms of patient engagement and well‐being.

Recurring structured assessments are increasingly used in cancer care to identify patient support needs [20]. In Swedish cancer care the Assessment of Rehabilitation Needs Checklist (ARNC) [10] is recommended by the Confederation of Regional Cancer Centres [21] as an initial screening tool to detect symptoms, signs and possible need for rehabilitation (see Supporting Information for full Swedish version). It covers 19 to 21 domains, depending on version, including physical, psychological, social and existential aspects. Patients rate their needs using a four‐point response scale. A follow‐up consultation is recommended, focusing on the patient's health problems and appropriate rehabilitation efforts. Despite national endorsement, the ARNC has not been studied in specialised settings such as allo‐HCT, where patient needs shift over time and nursing continuity is essential [22, 23]. Its routine clinical use and contribution to operationalising PCN remain unexplored. To explore this, the present study applies the Gothenburg Centre for Person‐Centred Care (GPCC) model [24], which defines person‐centred care through three interconnected components: (1) the patient's narrative, (2) partnership, and (3) a shared care plan. These components offer a basis for examining how PCN is provided in clinical interactions where the ARNC is used.

The aim of this study was to investigate how the use of ARNC as a conversation tool promotes person‐centred nursing (PCN) within the allo‐HCT context.

## 2 Method

### 2.1 Design and Study Context

This study is part of a larger research project (aCent), which focuses on developing, implementing and evaluating a model for person‐centred, systematic, nurse‐led support interventions in allo‐HCT care.

The present study employed a qualitative triangulation design, using multiple methods [19, 24, 25, 26] to explore PCN through the use of the ARNC and follow‐up conversations from different perspectives. The overarching project is conducted at two of Sweden's major allo‐HCT centres, selected for their high volumes of patients treated with allogeneic transplantation. Both inpatient and outpatient settings are included. The present study focused on gaining a broader understanding of how the ARNC was experienced and used. At the start of the overarching project, the ARNC was not routinely used. Therefore, before data collection began, RNs at both sites received structured training in person‐centred care and use of the ARNC. The ARNC had been introduced in practice prior to the study and key RNs acted as “champions” to support and facilitate the implementation process.

### 2.2 Sampling and Data Collection

Eligible patients were adults (≥ 18 years), Swedish‐speaking, without cognitive impairment and planned for allo‐HCT. Recruitment took place consecutively from March to September 2023 at the two largest Swedish HCT centres. Of 65 patients approached, 29 were excluded: 17 declined, 6 due to administrative issues, 4 non‐responders, and 2 due to transplant changes. The final sample was 36, of whom 16 were subsequently invited to interviews. RNs with experience of using the ARNC were invited to contribute through focus group interviews (FGI) and written memos. RN recruitment was by convenience sampling. All participants were approached in person or by phone and received written study information.

Three researchers conducted 16 telephone interviews using an interview guide designed to explore the patient experience of conversations involving the ARNC with open‐ended questions such as: “Tell me how you experienced filling out and using the ARNC as a basis for conversation with the RN?”. Interviews lasted 17–135 min (median 40 min) and were audio‐recorded, transcribed verbatim and anonymised. The interviews were conducted approximately 6 weeks after transplantation, once the patient had been discharged home.

Three FGIs were conducted with 16 RNs by three researchers. Two FGIs were from inpatient care, one from each site, while the third FGI included “champions” from both sites with experience in both inpatient and outpatient care. Two focus groups were conducted digitally and video‐recorded, while the third took place in person and was audio‐recorded. Each 50‐min session followed a semi‐structured guide exploring ARNC in clinical conversations using open‐ended questions such as “Describe how you perceived using the ARNC as a conversational tool in clinical care?” A researcher was present as an observer. Data collection continued until thematic saturation was reached, indicated by the recurrence of content and diminishing emergence of new themes across interviews and groups.

In addition, 30 memos were written by RNs immediately after using the ARNC. The memos, produced for research purposes, included brief notes about its use in practice, reflections on patient interactions and professional perspectives. Memos were collected during 2022, while interviews and FGI were conducted from June to December 2023.

The total volume of transcribed material amounted to approximately 260 pages. Researchers had no prior relationship with participants. Transcripts were not returned for comments or correction, although participants were informed they could request access. In clinical practice, the ARNC was completed just before the follow‐up conversation with the RN, either immediately or within a few days.

### 2.3 Data Analysis

**FIGURE 1:**
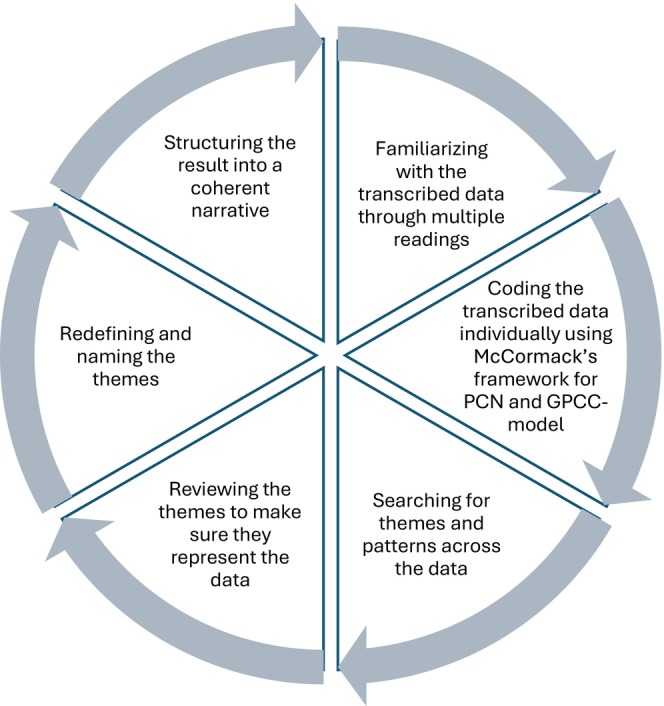
Overview of the reflexive thematic analysis process. The six iterative phases followed Braun and Clarke's reflexive thematic analysis [25, 26]. The analysis was adapted to reflect the Person‐centred nursing framework by McCormack and McCance [19]. In addition, the three components of the GPCC model [24] (the patient's narrative, partnership and a shared care plan) were explicitly applied as an interpretive lens to connect data‐driven codes and themes with theoretically grounded person‐centred dimensions.

Data were analysed using reflexive thematic analysis in six phases as described by Braun and Clarke [25, 26]. The process (Figure 1) began with repeated readings by two authors, who independently noted impressions and discussed them with a third author. As part of the familiarisation phase, one patient interview and six memos were excluded in line with reflexive thematic principles, due to limited narrative richness and relevance to the research aim.

Inductive coding was carried out separately by two of the authors within each data type. The analysis was conceptually informed by the PCN framework and the GPCC model [24]. While coding was primarily inductive the three GPCC components—the patient's narrative, partnership and a shared care plan—were used as an interpretive lens to connect data‐driven patterns to theoretically grounded person‐centred dimensions. The codes were compared across sources to identify patterns and generate preliminary themes. At this stage, all data sources (interviews, focus groups and memos) were treated as a single dataset, enabling cross‐data analysis to identify shared themes and codes. These themes were reviewed and refined through collaborative discussions between two authors and further adjusted in dialogue with a third author. Themes were defined and labelled to capture their interpretive meaning, while anonymised quotations were selected to illustrate descriptions of PCN. These choices were reviewed and discussed between three authors to ensure alignment with the study aim. Using researcher triangulation, the other authors reviewed and validated the emerged themes in the final stage.

Author contributions are presented in accordance with the CRediT taxonomy (Contributor Roles Taxonomy) at the end of the manuscript.

## 3 Results

### 3.1 Participant Characteristics

The study included interviews with 16 patients who had undergone allo‐HCT, FGIs with 16 RNs working in haematology care and 30 memos written by RNs, Table 1. Some of the RNs from the FGI also contributed to the written memos. For the total patient cohort (*n* = 36) demographic characteristics are summarised in Table S1.

**TABLE 1:** Participant characteristics.

Patients (*n* = 16)	
Age, median (range)	61 years (19–73)
Sex, *n* (%)	Male: 11 (69%) Female: 5 (31%)
Site, *n* (%)	Site One: 9 (56%) Site Two: 7 (44%)

RNs (*n* = 16)	
Focus Group 1	4 RNs (inpatient, Site 1)
Focus Group 2	5 RNs (inpatient, Site Two)
Focus Group 3	7 RNs (mixed in‐/outpatient, both sites)
Additional contributions	30 written memos submitted by RNs
Participant overlap	Some RNs contributed to both memos and FGI

### 3.2 Themes

The analysis generated three overarching themes: (1) *Letting the Story Emerge*, (2) *Unmet Needs*, and (3) *Structural Gaps in Practice*. Theme 1 included three subthemes, Theme 2 included none, while Theme 3 had two.

**FIGURE 2:**
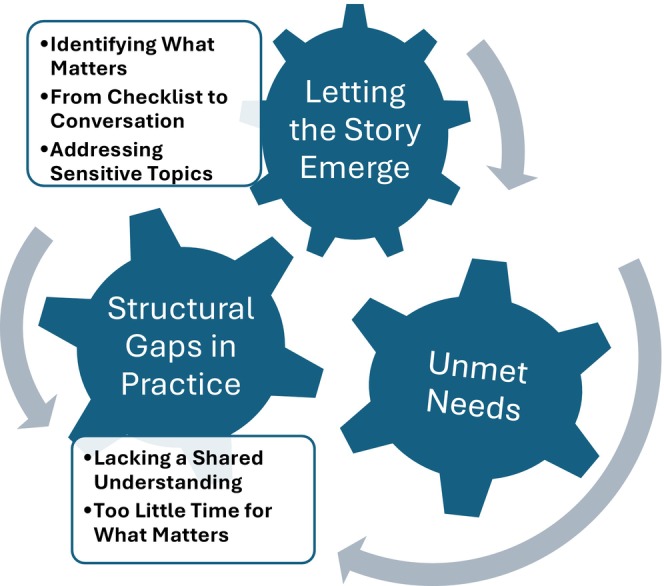
Thematic map of themes and subthemes.

These themes illustrate how the ARNC was used in clinical care and how PCN potential was shaped by relational and organisational conditions. The thematic map is presented in Figure 2.

The themes are presented below, each introduced by a brief summary capturing its core meaning.

### 3.3 Theme 1: Letting the Story Emerge

This theme highlights the ARNC potential to facilitate patient‐driven conversation, a core element of person‐centred practice.

#### 3.3.1 Subtheme 1.1: Identifying What Matters

Both patients and RNs expressed that the ARNC facilitated a more person‐centred approach by helping them identify each patient's individual needs. It enabled patients to bring their narrative to the forefront and define what was important, thus promoting patient participation. For some patients, it also served as a reflective tool, acting as a bridge to self‐reflection. Completing the ARNC helped them articulate and prioritise their needs by systematically reviewing their situation and identifying areas where they required additional information, help, or guidance.

It helps you put your feelings and thoughts into words. Now you have the facts in front of you. It makes it easier to talk about it. (Patient 5)

This theme indicates how the ARNC, when used as a conversational tool, enabled both patients and RNs to shift the focus from medical concerns to more individualised expressions of need. RNs expressed that the ARNC helped them to better understand patient's needs and allowed them to work in a more person‐centred way, revealing concerns that might otherwise have remained unspoken or unnoticed.

They expressed different needs that we would not have seen without the ARNC. I think it is a huge advantage to get help to work more person‐centred. (RN, Focus Group 1)

#### 3.3.2 Subtheme 1.2: From Checklist to Conversation

Some patients described using the ARNC as a mental checklist; for example, to prepare for a doctor's visit or to monitor their needs in daily life. However, both patients and RNs noted that the tool was most valuable when followed up by a conversation. In these encounters, the ARNC became more than an assessment, as it helped structure the conversation around the patient's needs. This supported more patient‐driven conversations and strengthened the partnership between patient and RN.

Talking to the nurse, it also feels good, that someone is going over things with you. I had a lot of anxiety and fear during the first few weeks. So, it helped me, for example, to talk about it when I filled in the form. (Patient 7)

Several RNs explained how the ARNC helped them step back from a checklist‐driven approach and let the patient take the lead, supporting more participatory conversations and patient reflection. Rather than setting the agenda themselves, RNs could allow the patient's own narrative to shape the conversation. One RN described how the tool changed the nature of the interaction, not merely a procedural change, but a shift in power and agency in the clinical encounter.

It becomes a completely different conversation. Instead of asking “Do you feel this or that?,” I ask: “Can you tell me from your perspective? How you experience it or don't experience it?” (RN, Focus Group 2)

According to the RNs, this way of working promoted a more collaborative relationship and facilitated greater involvement in care decisions.

#### 3.3.3 Subtheme 1.3: Addressing Sensitive Topics

A few patients emphasised how the ARNC helped address a broad range of concerns, including existential, financial and pain‐related concerns. Such concerns were often previously neglected.

Both patients and RNs expressed that using the ARNC broadened the perspective on patient needs and made it easier to discuss topics that were usually avoided, such as sexuality and financial concerns. These topics were rarely addressed, and patients often did not know they could bring them up.

It's like a kind of gateway, it makes it easier to talk about things in a different way, opening up the conversation. […] I guess a lot of people carry things around they never dare to ask about. (Patient 3)

Similarly, several RNs noted that using the ARNC facilitated discussions about family, relationships and financial difficulties, highlighting areas often overlooked in routine practice.

And with things like finances, for example, I can try to refer them to a counsellor who might be able to help with financial support and so on. I feel that using the ARNC has really helped with that. (RN, Focus Group 2)

The ARNC helped uncover new aspects of patients' stories and previously unspoken needs, such as psychological distress. Both patients and RNs emphasised that many were unaware of available psychosocial support. RNs described how they could guide the patient in identifying problems and exploring possible avenues for support. By making such needs visible, the ARNC enabled earlier referrals to healthcare professionals. This promoted interdisciplinary collaboration, a key aspect of person‐centred nursing.

### 3.4 Theme 2: Unmet Needs

This theme explores how patients' concerns sometimes failed to lead to continuity of care. While some patients described a fully person‐centred experience, many encountered a gap between what was shared and what happened afterward. In some cases, there was no conversation following the completion of the ARNC; in others, concerns were acknowledged but not addressed further. This lack of response affected patients' sense of trust, continuity and involvement in care planning.

Several patients described completing the ARNC but receiving no follow‐up, documentation or feedback. Some said they simply handed it in, not knowing if anyone had actually read it. For them, the ARNC felt like an administrative form rather than a meaningful part of their care. Others described sharing serious concerns but receiving no indication that anything would be done. As one patient reflected:

I don't really know what will happen if I write that I am suicidal. If I write that in the ARNC, will anyone see it? (Patient 1)

RNs noted that the ARNC was helpful for early identification of patient concerns, especially during admission, but acknowledged that there was not always a clear pathway for responding to these concerns. This created uncertainty for patients about whether their needs had been taken seriously.

Patients and RNs disagreed on whether the ARNC was helpful or led to nurse‐led interventions. Some patients explained that from their perspective, it was unclear whether the ARNC made any real difference. While they understood that RNs might use it to assess their well‐being, they did not know whether it truly helped them. Although patients often felt that the ARNC did not lead to concrete actions, RNs described how it frequently resulted in interventions. The RNs explained that the ARNC helped identify issues earlier, allowing them to refer patients to the appropriate healthcare professionals.

Both patients and RNs emphasised that lack of feedback or follow‐up limited the effectiveness of care. Even when concerns were raised and discussed, the absence of continuity reduced the likelihood that support would be sustained.

You want to give something back to the patient. Make them feel that they haven't just opened up there in the admissions office and told us about a problem… and then you basically just wave it away. And then they don't get any follow‐up on it later. (RN, Focus Group 2)

This theme illustrates that while the ARNC can prompt meaningful sharing, it does not automatically lead to action. The disconnect between what is expressed and what is acted upon may weaken the sense of continuity and partnership that person‐centred nursing aims to support.

### 3.5 Theme 3: Structural Gaps in Practice

While Theme 2 focused on unmet concerns in individual encounters, this theme addresses organisational conditions that hindered the ARNC's intended use for PCN in practice.

#### 3.5.1 Subtheme 3.1: Lacking a Shared Understanding

The purpose of the ARNC was often unclear. The level of information and education that patients and RNs had received varied, and for some, it was not evident that it was meant to be more than a checklist. Some patients questioned whether it was for their benefit or just for statistics, raising concerns about transparency.

When I filled it in, I thought, is this for my sake so that you will do something to make me feel better, or is it just for statistics? (Patient 6)

Another patient reflected similarly:

When I looked at the form, I couldn't really grasp what the purpose behind it was. Was it to improve care or to explore my situation? I had no sense of direction when responding. (Patient 4)

Such uncertainty appeared to shape how patients related to the form and whether they saw it as meaningful. On the other hand, the ARNC made little difference for patients who were already highly self‐reliant. These patients routinely raised concerns themselves and felt the ARNC added little to their care. One patient noted that they had always been health‐conscious, so completing the ARNC did not change their usual approach.

The ARNC was used in both in‐patient and out‐patient settings, but both patients and RNs described a lack of structure in how it was applied. Some patients mentioned struggling to differentiate it from other healthcare visits. They explained that they were already undergoing multiple assessments and follow‐ups, making it difficult to distinguish what the ARNC added. This suggests a lack of shared understanding, which may have undermined the ARNC's ability to support consistent person‐centred practice.

#### 3.5.2 Subtheme 3.2: Too Little Time for What Matters

The lack of structure when using the ARNC emerged as a key challenge for ensuring follow‐up, indicating organisational shortcomings in person‐centred nursing. Both RNs and patients emphasised that time constraints often prevented them from following up. They agreed that structured follow‐up in the form of re‐assessment would be beneficial, allowing for an understanding of how patients' needs evolve over time. However, RNs noted that using the ARNC as a conversation tool with follow‐up was time‐consuming, and structured re‐assessments were rarely prioritised. They explained that the assessment process itself could be lengthy, while the pressure to complete it quickly did not allow for meaningful engagement.

RNs emphasised that completing the ARNC was only the first step; it needed to be followed by a meaningful conversation. However, they described how these follow‐up conversations were often deprioritized in practice, particularly during shifts where time constraints made deeper conversations difficult, such as at night.

It's not just about handing in this form, but you have to have this follow‐up conversation as well, and then it's not really a priority to have that conversation in the middle of the night. (RN, Focus Group 2)

In these situations, follow‐up was sometimes deprioritized in favor of practical demands. Another challenge that patients and RNs identified was the transition between inpatient and outpatient care. They observed that collaboration between these settings was often limited, making continuity of care difficult. Some patients wanted a more structured approach, such as receiving a copy of the ARNC to bring to outpatient visits for follow‐up discussions. RNs also saw the potential benefit of a standardised framework to ensure that no patient concerns were overlooked during care transitions.

It is good to have something standardized, which everyone follows, so that nothing falls through the cracks. (RN, Focus Group 3)

These accounts illustrate how organisational conditions, such as time pressure, unclear routines and fragmented care, can disrupt the continuity that person‐centred nursing relies on.

## 4 Discussion

The aim of this study was to investigate how the use of the ARNC as a conversation tool promotes PCN within the allo‐HCT context. The findings indicate that the ARNC can generate meaningful conversations, reveal needs and contribute to shared decision‐making. However, significant challenges persist, particularly around follow‐up and the inconsistent use of the ARNC in clinical practice.

When used as part of a structured conversation, the ARNC can help identify needs that patients might not otherwise express. This aligns with McCormack and McCance's [19] emphasis on the patient's story as central to care. Friesen‐Storms et al. [27] similarly show how structured tools can create space for conversations that reveal hidden needs. By inviting discussions on emotional or existential aspects, the ARNC contributes to a more holistic understanding of the patient's situation, which is in accordance with recent studies on mental health in cancer care [28, 29, 30]. Melander et al. [31] further confirm that incorporating patient narratives into care improves rehabilitation outcomes.

Studies [27, 32, 33] show that structured assessments risk becoming mechanical tasks if they lack genuine engagement with the patient's story. To enable person‐centred approaches, the ARNC must lead to real conversations about the patient's situation, not just ticking off a checklist. Partnership and planning, which are central elements of PCN [19, 24, 34], require that conversations lead to shared care plans that reflect the patient's individual needs and preferences. This type of collaboration is demonstrated in the study by Melander et al. [31], where active patient involvement in planning contributed to more meaningful and responsive care. In the present study, however, while patients' needs were identified, integration into ongoing care routines remained limited, suggesting that conversations do not consistently translate into shared plans or concrete actions. One possible reason is the relatively recent implementation of the ARNC. Although RNs received initial training and support from local champions, the ARNC had only recently been introduced at the time of data collection. Establishing new routines takes time and requires both repetition and a supportive structure; without clear responsibilities and reinforcement, even useful tools may fail to achieve their full potential.

A related issue was the difference between conversation and action, which manifested differently between patients. Some were unsure whether their input led to changes in care. Others found the ARNC less meaningful as they had no further needs. This may reflect a high level of self‐management or the fact that allo‐HCT involves intensive care and frequent contact with RNs, especially during the early inpatient phase. In such settings, the ARNC may have appeared redundant. While some patients view the tool as unnecessary due to their own proactive approach, this variation demonstrates the need for equitable, not merely equal, implementation. Structured tools like the ARNC may be most beneficial for those less likely to articulate concerns independently, making it important that all patients are offered the opportunity, regardless of whether or not they actively request it.

These findings suggest a broader systemic issue: supporting PCN in clinical practice requires more than the introduction of structured tools. Although national guidelines [20] promote structured assessments, the practical support for integration into clinical routines is often lacking. Studies have shown that time constraints, unclear responsibilities and missing documentation routines can hinder continuity and shared planning [35, 36, 37, 38]. This emphasises the need for consistent routines to ensure continuity and clearer documentation pathways. In this study, the ARNC was primarily used to identify needs, but less often as a basis for care planning or structured follow‐up. There were no clear routines for documentation or shared care plans, which made it difficult to address identified needs over time. The gap between what is expressed and what is acted upon limits the ARNC's ability to support PCN in practice.

Assigning responsibility for maintaining competence over time is essential. To support PCN, RNs need training that covers both the technical use of the ARNC and the relational aspects of PCN. Sustained implementation requires not only initial training, but also repeated education, regular review and clear routines for ensuring that newly employed RNs are included. Individual commitment must be backed by clear expectations and consistent support from frontline and senior leadership. For structured conversations to become part of everyday care, the organization must take a share of the responsibility for making this possible. Effective PCN depends on both individual competence and organisational structures [33]. If structured assessments are part of national strategies, healthcare organisations must also ensure the conditions for their meaningful use. This echoes findings by Howell et al. [38], Melander et al. [31] and O'Sullivan et al. [39], who underline that implementation requires integration into daily routines and consistent organisational support.

Finally, the conversation prompted by the ARNC can itself be seen as an intervention: a moment where PCN begins to take shape. Some needs may be addressed directly, while others require follow‐up. Without supportive routines and shared responsibility, this potential remains limited. This study shows that the ARNC can support PCN when used as a space for meaningful conversation, but its contribution depends on whether patient concerns are acknowledged, acted upon and carried forward in care planning.

### 4.1 Limitations and Methodological Considerations

The authors bring both clinical and academic perspectives, including RNs with extensive experience in allo‐HCT care, person‐centred practice and qualitative research. This professional background contributed to a contextual understanding of the clinical setting and the relational dynamics central to nursing. At the same time, reflexivity was maintained throughout the study by critically examining how the researchers' own assumptions, values and prior experiences may have influenced the analytic process and the development of themes. Reflexive thematic analysis acknowledges that researchers' perspectives shape interpretation [25, 40, 41]. To ensure credibility, the team resolved discrepancies collaboratively, inspired by Nowell et al. [42]. This approach promotes transparency while recognising that researchers co‐construct meaning with participants.

Using multiple data sources and both patient and RN perspectives enabled triangulation and a richer understanding of the phenomenon. Triangulation can enhance credibility and reduce the risk of bias from single data sources [43].

The study was conducted at two major allo‐HCT centres in Sweden and its findings are closely linked to this specific context. While not generalisable, the insights may inform future use of person‐centred tools in cancer care. Lack of follow‐up limits assessment of sustained outcomes, especially psychological well‐being.

From a gender perspective, the patient interviews included more men than women, reflecting differences in availability during treatment. Patient characteristics, including clinical trajectory and care setting may have influenced how the ARNC was experienced. Perspectives from caregivers and the multidisciplinary team were not included. Given their established role in recovery [6], this is an important limitation. In addition, the study did not address potential challenges in using the ARNC among patients with cognitive impairment, language barriers, or limited health literacy.

Braun and Clarke [40, 41] stress the need for flexibility and reflexivity in qualitative research. This study applied reflexive thematic analysis, which allowed for inductive insights while remaining informed by theoretical frameworks. Researcher positioning was both a resource and a potential limitation. As highlighted by Koskinen [44], acknowledging researcher vulnerability and context strengthens the credibility and trustworthiness of qualitative findings. Findings should be viewed as context‐sensitive, not universally applicable. While not representative of all allo‐HCT patients, the sample captured variation in care experiences and the methodology enabled rich, practice‐relevant insights into how structured tools like the ARNC function in everyday nursing.

Future studies should include perspectives from family caregivers and interdisciplinary healthcare professionals to expand the understanding of how the ARNC supports holistic PCN. Longitudinal research is also needed to explore whether the ARNC has long‐term effects on patient well‐being and person‐centred outcomes. Additionally, research should address how the ARNC can be adapted for use with more diverse patient groups, including those with communication or cognitive barriers.

## 5 Conclusion

This study shows the potential of the ARNC to facilitate PCN in allo‐HCT care by providing a structured framework for patient‐driven conversations. Its success depends on the relational and organisational conditions in which it is used. To maximise the ARNC's potential, healthcare systems must ensure consistent follow‐up, clear documentation pathways and support for RNs to engage in meaningful, ongoing conversations with patients. By so doing the ARNC can become not just a tool for assessment, but for co‐creating care plans that reflect the individual needs and priorities of patients undergoing allo‐HCT.

## Author Contributions

C.E.de.C., A.O., A.M.K.: methodology, formal analysis, writing: original draft and review. C.L.H., J.W., A.M.K.: conceptualization, supervision. A.O., C.L.H., J.W., K.B.: investigation. C.E.de.C.: visualisation. All authors: validation; final approval of the manuscript.

## Ethics Statement

Ethical approval was obtained from the Regional Ethical Review Board in Stockholm, Sweden (No. 2022‐03688‐01). The study followed the Declaration of Helsinki and the research adhered to data confidentiality regulations. Participants were informed about the study, their right to withdraw and that participation was voluntary and anonymous.

## Conflicts of Interest

The authors declare no conflicts of interest.

## Supporting information


**Appendix S1:** Supporting Information.

**Appendix S2:** Supporting Information.

**Table S1:** scs70153‐sup‐0003‐TableS1.docx.

## Data Availability

The data that support the findings of this study are available on request from the corresponding author. The data are not publicly available due to privacy or ethical restrictions.
