# Supplementary material for: Person‐Centred Nursing in Allogeneic Stem Cell Transplantation Using a Conversation Tool: A Qualitative Study
Source: Scand J Caring Sci. 2025 Nov 7;39(4):e70153. doi: 10.1111/scs.70153 (PMC12592973; doi:10.1111/scs.70153)
Supplement: Supplementary file 2 — Appendix S2: Supporting Information. [file SCS-39-0-s003.pdf]

Hälsoskattning för cancerrehabilitering

Datum:……………………Namn:………………………………………………………………………………

Personnummer:………………………..Diagnos:..………………………………………………………………

Har din cancersjukdom påverkat nedanstående områden? Din beskrivning kan underlätta att du får rätt rehabilitering. Sätt kryss i den ruta som bäst motsvarar din upplevelse.

	Inget problem	Litet problem	Besvärande problem	Mycket besvärande problem	Kommentar
Trötthet	☐	☐	☐	☐	
Sömn	☐	☐	☐	☐	
Smärta	☐	☐	☐	☐	
Andning	☐	☐	☐	☐	
Minne/koncentration	☐	☐	☐	☐	
Nedstämdhet/ depression	☐	☐	☐	☐	
Oro/ångest	☐	☐	☐	☐	
Äta/dricka	☐	☐	☐	☐	
Illamående	☐	☐	☐	☐	
Stickningar i händer/ fötter	☐	☐	☐	☐	
Avföring	☐	☐	☐	☐	
Urin	☐	☐	☐	☐	

Vänd – flera frågor på nästa sida

HsC v2 © 2016, O-Nevo/Alkebro, UFC/USÖ, Box 1324, 703 13 Örebro. All rights reserved

Har din cancersjukdom påverkat nedanstående områden? Din beskrivning kan underlätta att du får rätt rehabilitering. Sätt kryss i den ruta som bäst motsvarar din upplevelse.

	Inget problem	Litet problem	Besvärande problem	Mycket besvärande problem	Kommentar
Utseende	☐	☐	☐	☐	
Fysisk aktivitet	☐	☐	☐	☐	
Sexualitet	☐	☐	☐	☐	
Familj/relationer	☐	☐	☐	☐	
Existentiella funderingar [1]	☐	☐	☐	☐	
Ekonomi	☐	☐	☐	☐	
Arbete/sysselsättning	☐	☐	☐	☐	

[1] Existentiella funderingar: Funderingar om livet och döden

Andra problem eller frågor kan du skriva om här:

HsC v2 © 2016, O-Nevo/Alkebro, UFC/USÖ, Box 1324, 703 13 Örebro. All rights reserved
