# Supplementary material for: Person‐Centred Nursing in Allogeneic Stem Cell Transplantation Using a Conversation Tool: A Qualitative Study
Source: Scand J Caring Sci. 2025 Nov 7;39(4):e70153. doi: 10.1111/scs.70153 (PMC12592973; doi:10.1111/scs.70153)
Supplement: Supplementary file 3 — Table S1: scs70153‐sup‐0003‐TableS1.docx. [file SCS-39-0-s001.docx]

**Supplementary Material**

**Supplementary Table S1. Characteristics of the total patient cohort (n = 36)**

| **Characteristic** | **Patients (n = 36)** |
| --- | --- |
| **Sex, n (%)** |  |
| Women | 14 (39) |
| Men | 22 (61) |
| **Age at allo-HCT, median (range)** | 55 (19–77) |
| **Education, n (%)** |  |
| Lower education | 13 (42) |
| Higher education (College/University) | 18 (58) |
| **Living situation, n (%)** |  |
| Living with someone | 22 (71) |
| Living alone | 7 (23) |
| Missing | 2 (6) |
| **Children under 18, n (%)** |  |
| Yes | 9 (29) |
| No | 21 (68) |
| Missing | 1 (3) |
| **Country of birth, n (%)** |  |
| Sweden | 26 (84) |
| Other | 5 (16) |
